# A Glyoxal Based Co-Condensation Adhesive with Excellent Water Resistance Using Chitosan and Starch as Enhanced Agents

**DOI:** 10.3390/polym18070853

**Published:** 2026-03-31

**Authors:** Jiawei Li, Lele Lu, Liangjun Xiao, Hui Wang

**Affiliations:** 1 Yunnan Provincial Key Laboratory of Wood and Bamboo Biomass Materials, Southwest Forestry University, Kunming 650224, China; lijiawei20000101@163.com (J.L.);; 2 Yunnan Academy of Forestry and Grassland, Kunming 650201, China

**Keywords:** wood adhesive, chitosan, starch, water resistance, plywood

## Abstract

To address the hazards posed by formaldehyde emissions from wood-based products to human health and the indoor environment, research on wood adhesives has focused on developing green and eco-friendly alternatives. However, the limited water resistance and bonding strength of bio-based or glyoxal-based adhesives have hindered their practical application. In this work, a co-condensation method was employed to prepare glyoxal-based co-condensation adhesive incorporating starch and a small amount of chitosan as synergistic reinforcing agents to enhance their cross-linking extent. Considering cost control, the starch content was varied to adjust the adhesive properties. When the molar ratio of glyoxal to urea was 2:1 and the mass ratio of starch to urea was 0.5:1, the adhesive exhibited optimal bonding strength, reaching 1.48 MPa after immersion in cold water for 24 h and 0.91 MPa after treatment in 63 °C hot water for 3 h. These values exceeded the requirements of the Chinese national standard (GB/T 9846-2015, ≥0.7 MPa). Structural analysis indicated Schiff base and aldol condensation reactions among amino groups in chitosan and urea and hydroxyl and aldehyde groups in starch and glyoxal, forming chemical covalent cross-links that contributed to improved water resistance and bonding strength of plywood samples. Furthermore, the excellent penetration ability of the adhesive could promote the formation of a uniform and dense cross-linked network under hot-pressing conditions, thereby enhancing the overall performance of the plywood.

## 1. Introduction

Formaldehyde-based resins, including urea-formaldehyde (UF), phenol-formaldehyde (PF), melamine-formaldehyde (MF), and their modified resins, have long been the dominant adhesives in the wood-based composites industry due to their excellent performance and low cost [1]. However, formaldehyde emissions have raised significant concerns regarding indoor air quality and environmental safety [2,3]. Consequently, bio-based adhesives derived from natural materials have gradually become a focus of research [4]. As reported in the literature, many biomass-derived polymers [5], such as soybean [6,7], tannin [8,9], lignin [10,11], wood fiber [12,13], plant-derived polymers and starch [14,15,16,17], possessed many advantages, including renewability, biodegradability, low toxicity, and abundant reactive functional groups (e.g., -OH and -NH_2_). These outstanding characteristics not only facilitate the construction of covalently cross-linked networks with aldehyde-based crosslinkers, but also enhance interfacial interactions with wood substrates through hydrogen bonding and mechanical interlocking [18].

As the second most abundant natural polysaccharide polymer in nature [19], starch has been widely utilized in the development of wood adhesives [20]. However, its limited bonding strength and water resistance have restricted its broader applications [13]. To maintain the characteristics of green and renewable, it was considered as an effective way to blend different biomass materials to achieve functional complementarity and enhance the performance of the resins [21,22]. Accordingly, in this work, chitosan, a natural polysaccharide rich in active amino and hydroxyl functional groups, was commonly used as an effective natural modifier to improve the performance of resins, particularly their water resistance [23]. Therefore, using chitosan as a modifier for starch-based adhesives was considered a feasible strategy to enhance bonding strength and water resistance [24,25,26]. However, starch–chitosan resins prepared via simple blending still failed to meet practical requirements in terms of water resistance [27]. Residual free hydrophilic groups in the cross-linked system could cause the adhesive to swell, disperse, or hydrolyze in water, resulting in limited water durability [28,29]. Therefore, it remains crucial to establish a stable cross-linking network, primarily based on chemical bonding, to enhance the overall performance.

For the modification of polysaccharide-based resins, aldehyde-based cross-linking agents were considered effective for improving bonding performance via constructing a three-dimensional network structure through chemical linkages. As a highly reactive and low-toxicity dialdehyde compound, glyoxal could undergo polymerization reactions with both the hydroxyl groups of starch and the amino groups of chitosan, thereby enhancing the water resistance stability and mechanical strength of the resins through chemical cross-linking [30,31]. Moreover, it was also a feasible strategy to use glyoxal as a substitute for formaldehyde to prepare eco-friendly adhesives in addressing formaldehyde emission concerns. However, it has been reported that urea–glyoxal (UG) resins prepared under urea-formaldehyde-like processing conditions generally showed poorer performance than conventional formaldehyde-based resins, especially with respect to water resistance. This was primarily due to the formation of cyclic structures during the glyoxal–urea reaction, which suppressed the development of highly branched polymer networks and leads to insufficient cross-linking density upon curing, a wet shear strength of ≥0.7 MPa has not been achieved in earlier studies [32,33]. From the perspectives of chemical structure and environmental compatibility, a starch–chitosan polymer could serve as an effective cross-linking agent to increase the branching degree of glyoxal-based resins. Therefore, the synthesis of a copolymerization resin was proposed using chitosan–starch, urea, and glyoxal as raw materials. This approach aimed to enhance the cross-linking degree of the resin system, ultimately achieving excellent bonding strength and water resistance after curing [34]. Additionally, the synthesized resin was expected to exhibit notable eco-friendly characteristics, aligning with the sustainable development goals of wood adhesive technology.

To control the cost of the resins, copolymerization in this work will be conducted with starch content as a variable. The structural characteristics, curing behavior, bonding performance, and water resistance of the prepared resins will be systematically investigated. The findings of this work are expected to provide a technical basis for the development of high-performance and eco-friendly wood adhesives.

## 2. Materials and Methods

### 2.1. Material

Chitosan (BR, deacetylation degree ≥ 90%) was purchased from Shanghai Yuanye Bio-Technology Co., Ltd., Shanghai, China, Cassava starch (food grade) was brought from Huantai Yufeng Modified Starch Factory Co., Ltd., Zibo, China, Urea (AR) and a 40% glyoxal aqueous solution (AR) were supplied by Sinopharm Chemical Reagent Co., Ltd., Shanghai, China, Sodium hydroxide (AR) and acetic acid (AR) were obtained from Shantou Dahao Fine Chemicals Co., Ltd., Shantou, China, Poplar veneers with 2 mm thickness and 8–10% moisture content were provided by Hebei Zhiwei Veneer Factory, Langfang, China. Distilled water was prepared in our laboratory.

### 2.2. Preparation of Co-Condensation Adhesive

**Scheme 1:**
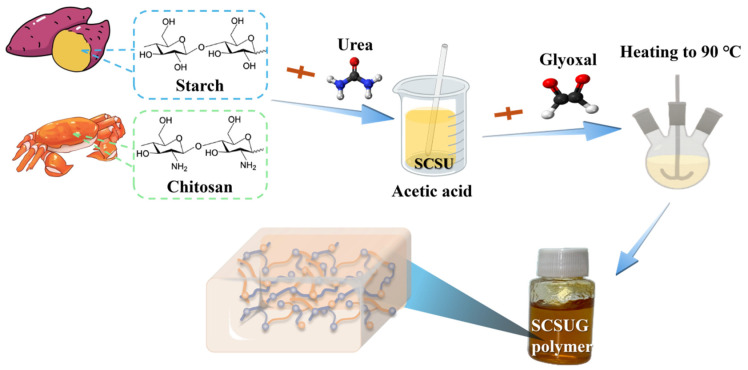
The preparation process of SCSU and SCSUG reins.

At room temperature, chitosan powder was gradually added to an aqueous acetic acid solution to prepare a homogeneous 2 wt % mixture. After complete dissolution, starch was added, followed by urea after 2 h under stirring according to the mass ratio shown in Table 1. The mixture was transferred into a three-neck flask equipped with a thermometer, mechanical stirrer, and condenser, a 40 wt % aqueous glyoxal solution was then introduced, with the molar ratio of glyoxal to urea maintained at 1:2. Subsequently, the pH of the mixture was adjusted to 8.0–8.5 using a 40 wt % aqueous sodium hydroxide solution, and heated in a water bath to 80 °C for 1 h. Afterward, the pH of the reaction system was readjusted to 5.5–6.5, followed by further heating to 90 °C and maintained for an additional 2 h. After cooling to room temperature, the resulting resin was named as SCSUG, the pH of the system was maintained in the range of 5.5–5.6, the acidic environment itself provides sufficient catalytic activity to promote the curing reactions during hot pressing, and no external curing agent was required. The formulations of the materials used are summarized in Table 1, and the general synthesis procedure is illustrated in Scheme 1.

### 2.3. Preparation and Performance of Plywood

The bonding strength of the synthesized resins was evaluated using three-ply plywood manufactured in the laboratory. The plywood was assembled from three poplar (Populus tremuloides) veneers, each 2 mm in thickness, following alternative grain orientation principles. Resin was applied to both surfaces of the core-layer veneer at a spread rate of 190 g/m^2^ using a brush. The assembled plywood was then hot-pressed at 150 °C under a pressure of 1.5 MPa for 6 min.

Based on the specifications of GB/T 9846-2015 [35], the plywood was cut into specimens with dimensions of 25 mm (width) × 100 mm (length). The specimens were randomly divided into three groups. One group was tested directly to determine the dry bonding strength, while the other two groups were subjected to water immersion prior to testing: one at (20 ± 3) °C for 24 h, and the other in hot water at 63 °C for 3 h. For each group, the bonding strength was calculated as the average value of at least six replicates.

### 2.4. Structural Characterization

#### 2.4.1. Fourier Transform Infrared Spectroscopy (FT-IR)

Fourier transform infrared (FT-IR) spectroscopy was employed to characterize the chemical structures of the resin samples. Solid samples of SCS, SCSU, and SCSUG were freeze-dried prior to test, while the cured SCSUG was obtained in an oven at 120 °C for 2 h. These solid samples were mixed with KBr at a mass ratio of 1:100 and then compressed into transparent pellets for analysis. The spectra were acquired using a Thermo Nicolet iS5 FT-IR spectrometer (Thermo Nicolet Corp., iS5, Madison, WI, USA) over a wavenumber range of 4000 to 500 cm^−1^, with a spectral resolution of 4 cm^−1^ and 32 scans. Liquid samples were analyzed under identical conditions using an attenuated total reflectance (ATR) accessory. All spectra were normalized to facilitate reliable comparison among different samples.

#### 2.4.2. X-Ray Photoelectron Spectroscopy (XPS)

The freeze-dried or cured resin samples was performed on an ESCALAB 250Xi spectrometer (Thermo Fisher Scientific, Waltham, MA, USA) equipped with a monochromatic Al Kα radiation source (hν = 1486.68 eV) to obtain their X-ray photoelectron spectroscopy (XPS) results. The accelerating voltage and emission current were 12.5 kV and 6 mA, respectively. All spectra were acquired under ultra-high vacuum conditions and processed using Avantage software (Version 6.8.1).

#### 2.4.3. X-Ray Diffraction(XRD)

X-ray diffraction (XRD) patterns of the freeze-dried and cured resin powders were recorded using a Rigaku SmartLab SE diffractometer (Rigaku, Tokyo, Japan) with Cu Kα radiation (λ = 1.5406 Å (angstrom)). The data were collected over a 2θ range of 5° to 90° at a scanning rate of 5°/min.

#### 2.4.4. Scanning Electron Microscopy (SEM)

The microstructures of the cured resin and bonded wood fracture surface were observed using a scanning electron microscope (SEM, S-3400N, Hitachi, Ltd., Hitachi, Japan). Prior to observation, all samples were sputter-coated with a thin layer of gold under vacuum to enhance surface conductivity. Imaging was performed at an accelerating voltage of 12.5 kV, and micrographs were acquired at various magnifications.

### 2.5. Residual Rate Test

The adhesive was dried to constant weight (M_0_) in an oven at 120 °C, wrapped by filter paper and immersed in water at (20 ± 3) °C for 24 h and in hot water at 63 °C for 3 h, respectively. After the water treatment, the residual samples were filtered and dried again at 120 °C to a constant weight (M_1_). According to Formula (1), the residual rate was calculated, and the final value was from the average of three parallel samples.

(1) $$\mathrm{Residual}\;\mathrm{rate}\;(\%)=\frac{M_{1}}{M_{0}}\;\times \;100\%$$

### 2.6. Curing and Thermal Properties of Resins

The curing characteristics of the resins was investigated using differential scanning calorimetry (DSC, NETZSCH, Selb, Germany). Approximately 5~10 mg liquid resin was sealed in an aluminum crucible and heated from 50 °C to 200 °C at a heating rate of 10 °C/min under a nitrogen atmosphere.

Thermal stability of the cured resins was evaluated using a thermogravimetric analysis instrument (NETZSCH TGA 209 F3, Selb, Germany). The samples were performed from 30 °C to 800 °C under a nitrogen atmosphere at a heating rate of 10 K/min.

### 2.7. Statistical Analysis

All analyses were performed on at least six independent replicates, and the data are presented as the mean ± standard deviation (SD). Statistical significance was evaluated using SPSS 24.0 software (IBM, Armonk, NY, USA), with differences considered significant at *p* < 0.01.

## 3. Results

### 3.1. The Bonding Strength and Water Resistance of SCSUG Resins

**Figure 1:**
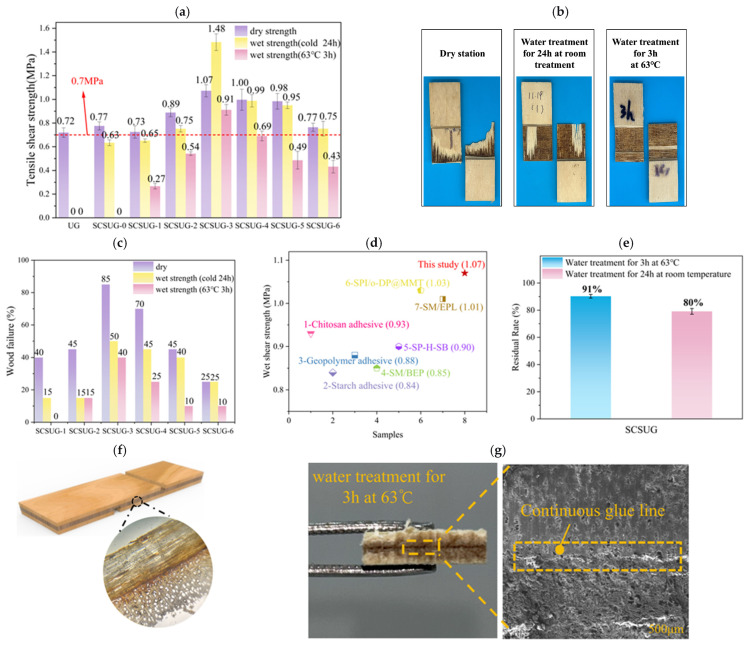
The bonding strength of resins (**a**), the fracture mode of plywood bonded with SCSUG-3 resin (**b**), wood failure of plywood bonded with SCSUG resins at dry, cold-water treatment and 63 °C hot-water treatment (**c**), rough comparison of wet bonding strength between SCSUG-3 and other modified bio-based adhesive (**d**), the SEM image of bonding line (**e**), the SEM image of bonding line after water treatment for 3 h at 63 °C (**f**), and residual rates of SCSUG-3 resin (**g**).

A series of co-condensation resins for wood bonding were synthesized by adjusting the starch content. The bonding strength was evaluated using three-layer poplar plywood. As shown in Figure 1a, comparing with UG resin, the bonding performance of the SCSUG adhesives exhibited a clear trend of first increasing and then decreasing with increasing starch content. The UG resin showed a dry bonding strength of 0.72 MPa, but almost no effective wet strength after immersion in cold water and 63 °C hot water, indicating that the cured UG resin possessed a relatively weak cross-linked structure and poor water resistance. When adding a little ratio chitosan, the SCSUG-0 resin showed a moderate improvement in dry and wet strength. However, its wet strength, especially after hot-water treatment, remained zero. This indicated that although chitosan can react with glyoxal in a chemical reaction to increase the cross-linking extent, the number of effective reactive sites in the system was still limited, resulting in the improvement of wet strength also being limited. Then, with the starch further being introduced into the system, the bonding strength increased significantly. It could be observed that all SCSUG resins met the dry strength requirement of the Chinese national standard (GB/T 9846-2015 [35], ≥0.7 MPa). However, the wet strength after both cold-water and hot-water soaking treatments initially increased and then decreased with increasing starch content, indicating that the starch content directly affected the adhesive strength between wood substrates. At relatively low starch contents, starch possibly acted as an effective reinforcing component due to its abundant hydroxyl groups, which actively participated in glyoxal-induced cross-linking reactions. This was beneficial for increasing the cross-linking density, thereby enhancing stress transfer at the wood adhesive interface and resulting in an improved bonding strength. However, excessive starch content introduced a large number of hydroxyl groups that cannot be fully involved in covalent cross-linking due to the limited glyoxal. As a result, an increased fraction of physically bonded starch domains was formed, which disrupted the continuity of the cross-linked network. Moreover, high starch content increased the viscosity of the adhesive and promoted molecular aggregation, reducing resin penetration into the wood substrate and weakening mechanical interlocking. The combined effects of reduced effective cross-linking density, network heterogeneity, and impaired interfacial penetration ultimately resulted in a decline in bonding strength. Therefore, when the mass ratio of chitosan and starch was 0.31:1, the resin (labeled SCSUG-3) exhibited optimal wet bonding strength. This suggested that the cured cross-linked network formed at this level provided the highest resistance to water. The failure modes of the tested samples (Figure 1b,c) further confirmed that the cured resin could form strong interactions between wood veneers. Moreover, compared with other resins reported in the literature (e.g., starch-, chitosan-, soy protein-based adhesives, and oligomer-based adhesives) [36,37], the synthesized resin in this work exhibited outstanding water resistance, as illustrated in Figure 1d.

Furthermore, taking SCSUG-3 resin as an example, after drying in an oven at 120 °C for 2 h, the residual rates of the cured resin following 24 h cold-water soaking and 3 h 63 °C hot-water soaking were 91% and 80%, respectively, as shown in Figure 1e. These results suggested that the cured cross-linked resin retained a certain degree of water solubility, which was likely due to the presence of hydrophilic groups in starch and chitosan. Moreover, this also demonstrated that, in addition to internal cross-linking, some free functional groups in the resin could react with the wood components to form a stable bonded network during the hot-pressing process. As shown in Figure 1f, the microstructure of the bonded layer revealed a continuous and intact glue line. Even after soaking in hot water at 63 °C for 3 h, the SEM image (Figure 1g) still displayed a dense cross-linked structure and a continuous glue line, indicating that the resin could penetrate into the wood cell lumina and form a strong cohesive bond under hot-pressing conditions. To further substantiate these observations at the chemical level, the structural characteristics of the resins would be analyzed and discussed in the following section.

### 3.2. Structural Characteristics

**Figure 2:**
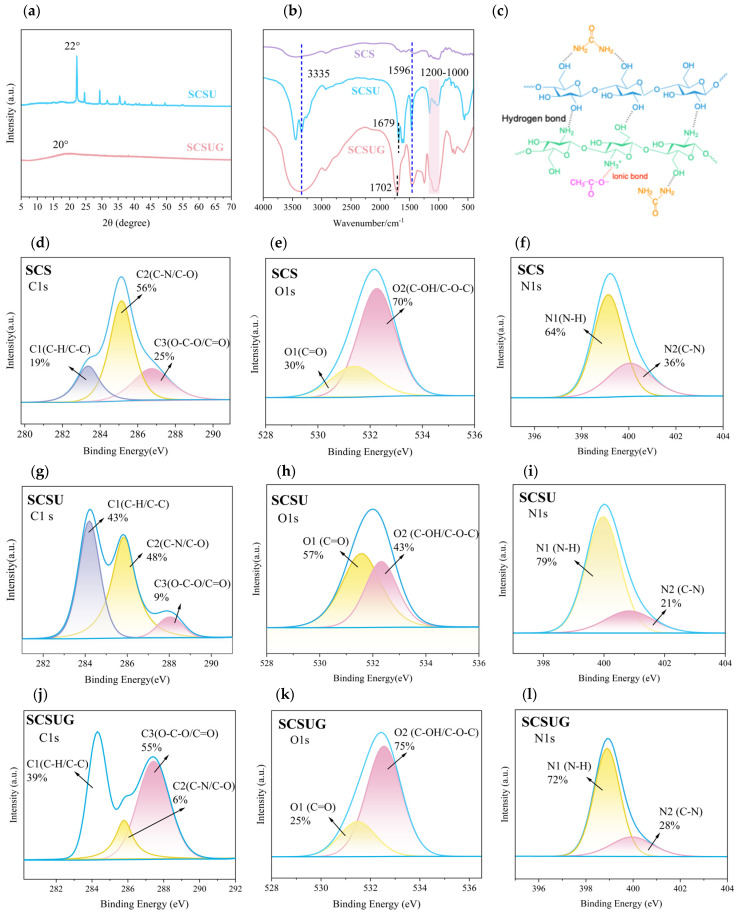

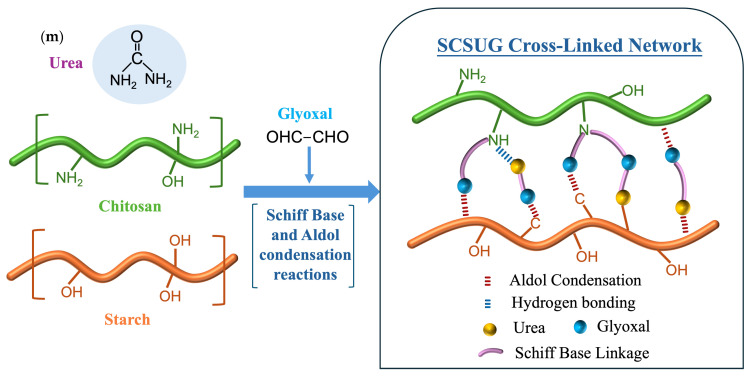
The XRD of SCSU and SCSUG resin (**a**), the FT-IR spectra of SCS, SCSU and SCSUG resin (**b**), a possible scheme of SCSU (**c**), the C1s of SCS resin (**d**), the O1s of SCS resin (**e**), the N1s of SCS resin (**f**), the C1s of SCSU resin (**g**), the O1s of SCSU resin (**h**), the N1s of cured SCSU resin (**i**), the C1s of SCSUG resin (**j**), the O1s of SCSUG resin (**k**), the N1s of SCSUG resin (**l**), and a possible scheme of SCSUG (**m**).

Based on the bonding strength results, the SCSUG-3 resin was selected as a representative sample to investigate its microstructural characteristics and the relationship with performance using FT-IR, XPS, and XRD. The corresponding results are presented in Figure 2. Theoretically, as a dialdehyde compound, glyoxal could undergo Schiff base and aldol condensation reactions with the amino groups in chitosan and the hydroxyl groups in starch under suitable conditions, forming stable amide bridges and acetal bonds. Additionally, urea could also react with glyoxal to generate urea-based bridging structures [29,34]. Thus, a cross-linked network could be established through the “bridge” role of glyoxal. Figure 2a shows the XRD patterns of the SCSU mixture and the SCSUG resin. In contrast to the SCSU sample, the XRD curve of the SCSUG resin displayed only a broad amorphous halo near 2θ ≈ 20° after reaction with glyoxal, indicating that the glyoxal disrupted the crystalline structure of the SCSU mixture. This was likely attributable to the formation of a cross-linked system. The structures of various samples were further analyzed by FT-IR, as shown in Figure 2b. By comparing the spectra of the SCS and SCSU samples, the appearance of a doublet peak near 3335 cm^−1^ indicated that different types of O-H and N-H vibrations were formed in the system after urea addition. The peak at 1679 cm^−1^ corresponded to amide I [38], and a sharp and strong absorption peak emerged following urea introduction, attributed to the stretching vibration of C=O of urea [28]. The peak at 1596 cm^−1^ was assigned to the bending vibration of N-H, and its intensity in the SCSU sample was higher than that in the SCS sample, also originating from the N-H bending vibration of urea [39]. These results suggested that a homogeneous solution was formed primarily through physical interactions among urea, starch and chitosan. Possible binding modes are illustrated in Figure 2c. Combined with the XPS results shown in Figure 2d–i and the assignment of peaks summarized in Table 2, the proportion of the C1 peak in the SCSU sample increased from 19% (SCS) to 43%, the O1 peak increased from 31% (SCS) to 57%, and the N1 peak (SCS) increased from 64% to 79%. These data provided further corroborative evidence that urea was incorporated into the polysaccharide system via physical interactions.

After the addition of glyoxal, the O-H and N-H stretching vibration peaks near 3335 cm^−1^ in the SCSUG resin became significantly broader. This could be partly attributed to the presence of moisture and partly to the homogenization of the chemical environments of the O-H and N-H functional groups, likely resulting from cross-linking with glyoxal. A newly emerged characteristic absorption peak at 1702 cm^−1^ could be assigned to the stretching vibration of the C=N bond, which was considered a characteristic feature of the imine structure formed via the Schiff base reaction between glyoxal and -NH_2_ groups [40]. Meanwhile, the substantial weakening of the absorption peak at 1596 cm^−1^, corresponding to free amino groups, further corroborated the involvement of amino groups in covalent cross-linking reaction. The enhanced absorption peaks in the 1200–1000 cm^−1^ region could be attributed to the vibrations of C-O-C and C-N bonds, suggesting that glyoxal also underwent acetalization reactions with the hydroxyl groups in starch, thereby promoting the formation of a cross-linked network. Although the chitosan content was relatively low, its role in the adhesive system was not primarily to act as the main cross-linking component, but rather as a highly efficient multifunctional macromolecular modifier, promoting the formation of a more homogeneous and mechanically robust interpenetrating cross-linked structure. Additional evidence could be obtained from the XPS results of the resins. As shown in Figure 2g–l, after reaction with glyoxal, the N1 peak ratio in the SCSUG resin decreased from 79% (SCSU) to 72%, further supporting the participation of N–H bonds in chemical cross-linking with glyoxal during resin synthesis [41]. The O1 peak ratio decreased from 57% (SCSU) to 25%, while the O2 peak ratio increased from 43% (SCSU) to 75%. This shift indicated the formation of additional C–O covalent structures via acetalization during the reaction with glyoxal. Additionally, the ratio of O2 peak of the SCSUG resin increased from 43% (SCSU) to 75%, and the N2 peak ratio increased from 21% (SCSU) to 28%. These results further confirmed the occurrence of chemical cross-linking reactions between glyoxal and both amino and hydroxyl functional groups.

Based on the above analysis, it could be concluded that glyoxal indeed functioned as a “bridge” molecule, as initially proposed, constructing a covalently cross-linked structural system through Schiff base and acetalization reactions. This provided a solid foundation for the excellent water resistance and mechanical strength of the resin. The proposed possible cross-linking scheme among the different raw materials in the SCSUG resin is illustrated in Figure 2m.

### 3.3. The Curing and Thermal Stability of the SCSUG Resin

**Figure 3:**
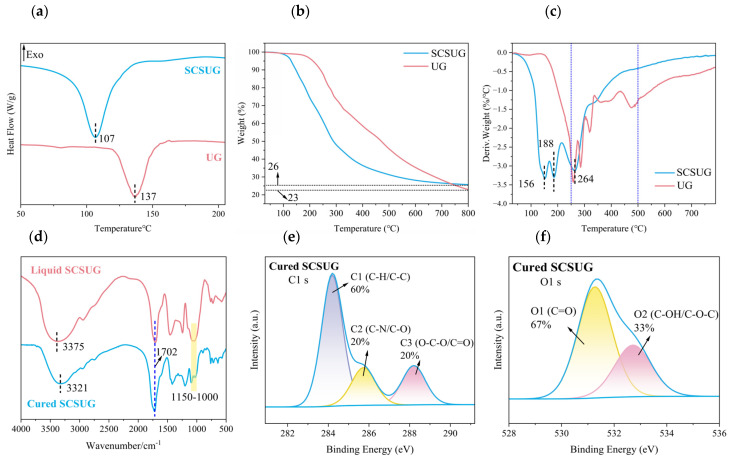
The DSC result of SCSUG and UG resins (**a**), the TG (**b**) and DTG (**c**) process of SCSUG resin, the FT-IR spectra of liquid and cured SCSUG resin (**d**), the C1s of cured SCSUG resin (**e**), and the O1s of cured SCSUG resin (**f**).

The curing characteristics of the SCSUG-3 resin were evaluated using DSC, and the results are shown in Figure 3a. The primary curing occurred between 100 °C and 140 °C, with a peak temperature of 107 °C. Compared with urea–glyoxal (UG) resin, the SCSUG-3 resin cured more readily under the same condition. The presence of a single curing peak indicated that starch, chitosan, urea and glyoxal in the SCSUG-3 resin formed a homogeneous system through physical and chemical interactions. Although the curing peak temperature was 107 °C, the hot-pressing temperature for plywood preparation was set to 150 °C to account for practical considerations such as heat transfer efficiency and heat loss.

The thermal stability of the cured SCSUG-3 resin was investigated by TG and DTG, and the results are shown in Figure 3b,c. The primary weight loss occurred between 100 °C and 400 °C. Weight loss below 100 °C was likely associated mainly with the evaporation of bound water within the resin. A noticeable weight loss between 100 °C and 220 °C may be related to the removal of residual functional groups that did not fully participate in the curing reaction. The weight loss between 220 °C and 400 °C was mainly attributed to the decomposition of the cross-linked network backbone. In particular, the degradation occurring at 264 °C was typically ascribed to the cleavage of covalent cross-links formed between glyoxal and amino groups. Compared with the UG resin, the SCSUG-3 exhibited a higher residual carbon rate (26%) due to the introduction of polysaccharide polymers, although its major weight loss was largely completed at relatively lower temperatures; the higher char residue indicated that the cross-linked system maintained improved structural stability at elevated temperatures after the main degradation stage. This behavior was mainly associated with the presence of polysaccharide backbones and the formation of a multi-point covalently cross-linked with glyoxal.

Furthermore, the structural characteristics of the SCSUG-3 resin before and after curing were analyzed using FT-IR and XPS, as shown in Figure 3d–f. Compared with the liquid resin, the broad peak at 3321 cm^−1^ in the cured SCSUG-3 resin exhibited reduced intensity and a slight redshift, indicating that O-H and N-H bonds underwent further cross-linking during the curing process as moisture evaporated, and that hydrogen-bonding interactions were also formed in the cured resin. The peaks at 1702 cm^−1^, corresponding to C=O (amide I band) and C=N (imine bonds), became sharper and more intense, suggesting that Schiff base reaction continued to promote the establishment of the cross-linked network during curing. Combined with changes in the C1s, O1s, and N1s peak ratios in the XPS spectra, these observations further corroborated the occurrence of Schiff base reactions between amino groups and glyoxal during the curing process, which was consistent with the structural changes observed during resin formation. The peaks between 1000 cm^−1^ and 1150 cm^−1^ corresponded to the stretching vibrations of C-O-C/C-O bonds (including those in polysaccharide backbones) [42]. These peaks decreased sharply with curing, which reflected the consumption of reactive functional groups during the high-temperature curing stage, indicating further cross-linking and glyoxal-induced cross-linking among polysaccharide chains. Simultaneously, compared with the liquid resin, the proportion of the C2 peak in the cured SCSUG resin increased from 6% to 20%, while the proportion of the O2 peak decreased from 75% to 33%. This further confirmed the role of Schiff base and acetalization reactions in reinforcing the cross-linked network with polysaccharide polymers. These findings also provided supports for the improved mechanical strength of the plywood and the increased residual carbon rate observed.

## 4. Conclusions

To develop an eco-friendly glyoxal-based wood adhesive to meet the performance requirements of the wood industry, a copolymerization strategy was adopted to construct a co-condensed adhesive reinforced with starch and a small amount of chitosan. When applied in the fabrication of three-ply poplar plywood, the adhesive exhibited optimal performance at a 0.5:1 mass ratio of starch to urea, achieving a dry bonding strength of 1.07 MPa, and a wet bonding strength of 0.91 MPa after immersion in 63 °C water for 3 h. These values fully satisfied the requirements for Type II plywood specified in GB/T 4897-2015 [43] and GB/T 9846-2015 [35].

Structural characterization demonstrated that starch, urea, and chitosan were chemically integrated into a cross-linked network, with glyoxal acting as an effective covalent bridging agent. After curing, the formation of a dense three-dimensional network endowed the bonded joints with excellent water resistance, as evidenced by the intact adhesive layer after hot-water treatment. Thermal analyses further confirmed that the resin possessed a relatively low curing temperature and good thermal stability, which were advantageous for industrial application.

Overall, this study provided a feasible strategy for the preparation of high-performance, glyoxal-based wood adhesives reinforced with natural polysaccharides. Nevertheless, from the perspective of large-scale application and sustainable development, future research should focus on further reducing the chitosan content and associated material costs, optimizing biomass resource utilization, and improving the economic competitiveness of the adhesive system. Addressing these challenges will be critical for promoting a high-value, sustainable supply chain for bio-based adhesives in the plywood industry.

## Figures and Tables

**Table 1:** Formulations of the SCSUG resins.

Resins	Chitosan:Urea (Mass Ratio)	Starch:Urea (Mass Ratio)	Glyoxal:Urea (Molar Ratio)
SCSUG-0	1:10	0:1	1:2
SCSUG-1	1:10	0.25:1	1:2
SCSUG-2	1:10	0.5:1	1:2
SCSUG-3	1:10	0.75:1	1:2
SCSUG-4	1:10	1:1	1:2
SCSUG-5	1:10	1.25:1	1:2
SCSUG-6	1:10	1.5:1	1:2

**Table 2:** XPS peak assignments of SCSUG-3 resin.

Spectral Region	Peak	Binding Energy (eV)	Chemical State	Assignment	Structural Implication
**C1s**	C1	284.8	C-H/C-C	Aliphatic carbon	Polysaccharide backbone
	C2	286.3	C-N/C-O	Ether or amine carbon	Starch, chitosan, urea
	C3	287.8	O-C-O/C=O	Acetal carbon	Glyoxal–polysaccharide cross-linking
**O1s**	O1	531.2	C=O	Carbonyl carbon	Urea and Schiff-base related structures
	O2	532.7	C-OH/C-O-C	Ether or amine carbon	Starch, chitosan, urea
**N1s**	N2	400.8	C-N	Imine nitrogen	Schiff-base linkage

## Data Availability

The original contributions presented in this study are included in the article. Further inquiries can be directed to the corresponding authors.

## Abbreviations

The following abbreviations are used in this manuscript:

FT-IR	Fourier transform infrared spectroscopy
XPS	X-ray photoelectron spectroscopy
XRD	X-ray Diffraction
SEM	Scanning electron microscopy
